# Listening to Australians with ovarian cancer: a cross-sectional survey investigating clinical trials awareness, information access and participation

**DOI:** 10.1007/s00520-026-10586-1

**Published:** 2026-03-22

**Authors:** Natalie Williams, Yeh Chen Lee, Hayley Russell, John Andrews, Won Sun Chen, Bridget Bradhurst

**Affiliations:** 1 Ovarian Cancer Australia, Melbourne, VIC 3000 Australia; 2 Curtin University, Bentley, WA 6102 Australia; 3 Australia New Zealand Gynaecological Oncology Group, Camperdown, NSW 2050 Australia; 4 Medical Oncology, Prince of Wales Hospital and Royal Hospital for Women, Sydney, NSW Australia; 5 School of Clinical Medicine, Faculty of Medicine and Health, UNSW Sydney, Sydney, NSW Australia

**Keywords:** Ovarian cancer, Clinical trials, Awareness, Gynaecological cancers, Ovarian neoplasms

## Abstract

**Purpose:**

To inform development of centralised, evidence-based clinical trials resources for Australians with ovarian cancer, a structured understanding of knowledge gaps and resource needs was essential. The study aimed to assist resource development by assessing awareness, information access, and participation in clinical trials of ovarian cancer patients.

**Methods:**

A national, cross-sectional online survey among Australians with ovarian cancer was conducted between October and November 2024. Descriptive and inferential statistics along with qualitative content analyses were conducted. Associations were examined using Chi-Square and Fisher Exact tests.

**Results:**

Surveys from 272 respondents indicated moderate knowledge ($\overline{x }$ = 4.46/10, SD = 2.34) and a high perception of importance of clinical trials ($\overline{x }$ = 9.27/10, SD = 1.17). 56% of respondents reported not receiving clinical trials information and 44% had sought information themselves. Respondents preferred information by email newsletter (34%), through health professional discussions (20%) and accessing an online information hub (17%). Information access enablers included clinicians being knowledgeable about clinical trials, personalised discussions, and access to a centralised information source. Barriers included fragmented information across websites, use of complex medical language and competing responsibilities as caregivers. A qualitative analysis of open-ended responses (*n* = 96) revealed three core themes: ‘we need better solutions to help find information and participate in clinical trials’, ‘weighing up options’ in their decision to participate, and ‘we want to help improve outcomes for women in the future’.

**Conclusions:**

Results from this study of Australians with ovarian cancer inform actionable change through development of evidence-based, tailored resources. Further solutions and evaluation of intervention effectiveness will continue through sector collaboration.

**Supplementary Information:**

The online version contains supplementary material available at 10.1007/s00520-026-10586-1.

## Introduction

Clinical trials in ovarian cancer are critical in the development of emerging drug therapy and interventions and can provide people with an opportunity to contribute to improving oncology care. In a disease frequently diagnosed at an advanced stage, a consequently high symptom burden, poor survival outcomes and considerable impacts on quality of life [1], timely awareness and access to clinical trials can be vital to improving recruitment [2]. Furthermore, improving trial awareness amongst people with ovarian cancer is recommended to improve access and participation in the context of shared decision-making [3].

Online resources detailing current clinical trials are available. However, many are aimed at clinicians, which can make the comprehension of information challenging for patients due to the use of medical terminology. Common barriers to trial participation and an acknowledgement of the physical and emotional impacts, factors which can affect decision-making, are lacking within information that appears to be available. In Australia, examples include the Australia and New Zealand Clinical Trials Registry (ANZCTR) and ClinTrial Refer, whilst ClinicalTrials.gov is the most prominent resource utilised internationally.

### International evidence of ovarian cancer trial awareness

Over recent years, increasing numbers of Australians with ovarian cancer are being told about, or offered to participate in, clinical trials [4]. However, the factors influencing clinical trials awareness or access to information were unknown. Previous international studies have reported varying degrees of awareness about cancer clinical trials (49–97%) in multiple tumour cohorts, though ovarian cancer patients were either not included or not reported as included in any of these [5–8]. Korean cancer patients’ awareness of clinical trials was quantified using scales of awareness and understanding. While most participants (97.4%) had heard of clinical trials, less than a quarter reported the two highest scales of understanding, with 5% able to explain clinical trials in detail and 18% in rough detail [7].

### Factors affecting trial information access and participation

Other studies have investigated factors influencing clinical trial information access and participation. In Australia, the decision to participate in a clinical trial was influenced by awareness in 88% of 162 health consumers [2]. Australian women with gynaecological cancers are known to be motivated to participate in research [9, 10]; however, less than 20% of those with ovarian cancer have reported participating in a clinical trial [4]. Factors such as trial availability and timing, eligibility restrictions, and communication of trial information may also be influencing participation rates [11, 12] and Australian women report feeling ‘left behind’ other cancer types with limited opportunities to participate in clinical trials [10].

Previous work investigating trial enrolment amongst underrepresented populations in the United States conceptualised the barriers and promoters of enrolment used within interventions [13]. These were categorised as factors affecting awareness, opportunity and acceptance/refusal of trial enrolment which were influenced by sociodemographic factors such as age, gender, geography and ethnicity [13]. Factors such as knowledge, attitudes, health literacy, eligibility, access, and altruism reflect the findings seen in more recent research on trial participation [10–12].

As ovarian cancer care rapidly evolves with advances in comprehensive genetic profiling and targeted therapies, it is more important than ever to ensure equitable trial access for those facing this high-mortality cancer; yet barriers persist [14]. A preceding qualitative research study undertaken in 2024 by this paper’s authorship team including 16 Australians with lived experience of ovarian cancer. Exploring their insights prior to the current study highlighted they are seeking credible, centralised information, trusted communication and tailored, relevant resources [10]. Furthermore, these women reflected that while they were willing to participate in clinical trials, barriers affect their awareness and participation and solutions are needed. While emotions regarding trials were varied, altruism was a motivator and they needed to self-advocate through instigating conversations with their clinical team and doing their own research [10]. Research to include a broader population sample was recommended to investigate information-seeking behaviours and preferred solutions thereby fulfilling an existing gap in evidence to inform the development of suitable information resources to meet the needs of the broad ovarian cancer population in Australia.

### Study aim and objectives

This study aimed to address the current knowledge gap by investigating clinical trials awareness, information access and participation amongst Australians with a lived experience of ovarian cancer. The study’s objectives were to:

1. Investigate perceptions on the role of clinical trials in ovarian cancer care;
2. Identify factors influencing information access about ovarian cancer clinical trials;
3. Identify factors contributing to ovarian cancer clinical trial participation;
4. Describe preferred sources of ovarian cancer clinical trials information; and
5. Explore how access to clinical trials information and participation for ovarian cancer could be improved.

## Methods

An exploratory sequential mixed methods design guided this investigation through a two-phased approach. This multi-phase approach was pre-planned to enable data integration in a sequential manner through the process of building [15]. Findings from the phase one qualitative descriptive study are reported elsewhere [10]. In phase two, a cross-sectional survey design sought to further meet the study aims through quantitative and qualitative data collection using survey questions, an accepted approach to study patient attitudes of health-related behaviours [16]. The philosophical paradigm of pragmatism underscored the methodology by collaborating with people with lived experiences to explore the development of solutions [17]. Reporting has been guided by the Strengthening the Reporting of Observational Studies in Epidemiology (STROBE) checklist [18] and the Checklist for Reporting Results of Internet E-Surveys (CHERRIES) [19] (available in supplementary file 1).

### Participants

Participants included a convenience sample of people with lived experience of ovarian cancer, aged 18 years or older, residing in Australia and diagnosed with ovarian cancer, or experiencing a disease recurrence, within 5 years. Ovarian cancer was inclusive in its definition as cancers originating in the ovary, fallopian tube, and/or peritoneum due to their similarities [20]. Inclusion criteria also required either proficiency in reading and writing English or having someone available to help with interpretation. Acknowledging the high incidence of ovarian cancer treatment impacting cognitive capacity, a telephone survey option was offered at online survey commencement, prior to consent; however, no participants took this option.

### Sample size estimation

Approximately 5000 Australians are currently living with ovarian cancer [21]. A target sample size of 257 participants was determined to achieve a confidence interval of 90% with a 5% margin of error.

### Survey instrument

In the absence of an existing validated survey tool to investigate clinical trials awareness, information access and participation, a purpose-designed survey was developed. Survey questions were developed to meet the study objectives. As recommended [22] in survey development, a review of existing tools from previous studies on clinical trials awareness and attitudes [3, 8] was undertaken, and relevant items were categorised by the study objectives. Further items were informed by results of the preceding qualitative study [10], and an expert stakeholder group, including people with lived experience of ovarian cancer. Recommended by Chi et al. [23], item development was also influenced by a conceptual framework [13] outlining factors influencing trial awareness, opportunity and acceptance/refusal of enrolment. Mapping these factors to developed items was undertaken to ensure comprehensive inclusion of influencing concepts along with items to examine sociodemographic factors and information sources.

Quantitative and qualitative data were collected across 91 items using self-reported ratings, Likert scales, multiple choice, and open-ended questions. Adaptive questioning was incorporated to reduce the number of relevant items based on previous responses. Combining these enhances methodological triangulation and enables respondents to provide broad descriptions of experiences and perceptions [24, 25]. The full survey tool is available in supplementary file 2.

Survey content validity was assessed by a group of four experts including clinicians and researchers. The content validity index (CVI) [26] was calculated to measure relevance, clarity, conciseness, and ambiguity of survey items across two rounds, with amendments made based on feedback after round one. All measures achieved a CVI of > 0.8 following round two. Additionally, the survey was piloted with 7 eligible participants. Feedback prompted minor changes to item syntax and formatting, and the pilot survey responses were included in final analysis.

### Recruitment and data collection

Recruitment occurred using multiple strategies from September to November 2024. The first saw distribution of the online survey via direct email to 2279 Australians with ovarian cancer from an electronic database of a non-government, not-for-profit organisation providing support and advocacy services (Ovarian Cancer Australia (OCA)). Next, OCA social media posts reached 37,000, 2000 and 700 users through Facebook, Instagram and LinkedIn channels respectively, and a webpage on the OCA website outlining the study and providing survey access was viewed 192 times during the recruitment period. Additionally, a recruitment letter was offered to eligible participants by doctors and nurses during ovarian cancer clinical appointments in two settings in New South Wales and two in Western Australia. Recruitment materials are available as supplementary file 3.

Data were collected via an open, voluntary, online survey using REDCap electronic data capture tools hosted at Curtin University [27]. Items were displayed across 13 pages with between 2 and 14 items per page. Participants could return to previous items using the ‘back’ button. As the survey was anonymous, information to identify duplicate entries was not collected. Consent to participate was collected via a compulsory hurdle question at the beginning of the survey. Data were collected from September to November 2024.

### Data analysis

Descriptive statistics, such as mean and standard deviation, are presented for continuous variables, with frequencies and percentages for categorical variables. A Chi-Square test or Fisher Exact test was used to examine associations between participants’ characteristics and responses. A *p*-value of < 0.05 was considered statistically significant for all 2-sided tests. In the instance where participants exited the survey before completion, case-wise omission was utilised for missing data, performed using IBM SPSS Statistics version 29 [28].

Responses to open-ended questions were analysed using an inductive approach to conventional content analysis [29]. Due to the sequential, exploratory approach, data were analysed considering the preceding qualitative investigation’s findings [10]. Data analysis used a reflexive process, following the steps: (1) data familiarisation; (2) dividing text into meaning units; (3) condensing meaning units; (4) labelling meaning units as codes; (5) grouping codes into categories describing who, what, when and where; (6) developing themes expressing how, in what way and by what means [30]. The primary author undertook initial coding on all open-ended responses using NVivo software version 14. This author is a female nurse researcher with 9 years of research experience and a clinical background in gynaecological cancer nursing. A second author undertook data familiarisation and simultaneous coding of a selection of responses to establish reliability. This author is a female counsellor and clinician researcher with 7 years of research experience and a clinical background in grief and loss, cancer care and palliative care. A table of themes and subthemes along with their corresponding codes, definitions and support quotations was systematically developed to ensure comprehensive inclusion of all data in the final results. Due to the anonymous nature of the survey, responses were not returned to participants, but were instead presented to two independent people meeting the survey eligibility criteria who confirmed them as an accurate representation of their experience.

### Ethical considerations

This study was conducted in accordance with Australian standards for the ethical conduct of research [31, 32], jurisdictional guidance for implementing the Declaration of Helsinki [33]. The Curtin University Human Research Ethics Committee (HREC) reviewed and approved the study (HRE2023-0637). Reciprocal approval was granted through the Child and Adolescent Health Service Low Risk Ethics Committee (RGS0000006962).

## Results

### Participant characteristics

Survey responses from 272 individuals who had experienced a diagnosis or recurrence of ovarian, fallopian tube, or peritoneal cancer within the past 5 years were included in the analysis. Details on participant characteristics are presented in Table 1.

**Table 1:** Survey participant characteristics

Characteristic	*n*	Valid percent (%)
Age (years) (***n*** = 272)		
Mean (standard deviation)	61.5 (10.40)	
Median (min-max)	63.0 (18–89)	
18–≤ 59	100	36.8
≥ 60	172	63.2
Location (***n*** = 272)		
Major city	229	84.2
Other (i.e. regional, remote)	43	15.8
State/territory (***n*** = 231)		
Victoria	72	31.2
New South Wales	64	27.7
Queensland	57	24.7
Western Australia	25	10.8
South Australia	10	4.3
Australian Capital Territory	2	0.5
Northern Territory	1	0.5
Tasmania	0	0.0
Gender (***n*** = 232)		
Female	232	100.0
Highest education qualification (***n*** = 232)		
Less than year 12 or equivalent	31	13.4
Completed year 12 of equivalent	18	7.8
Trade or technical certificate or diploma	57	24.6
University undergraduate degree	53	22.8
Postgraduate/higher university degree	73	31.5
Ethnicity (***n*** = 232)		
Australian (including Aboriginal Australian and mixed ethnicity such as British-Australian, Asian-Australian)	210	90.5
Other (i.e. Italian, New Zealander, Chinese, Middle Eastern, Punjabi, Vietnamese)	18	7.8
Prefer not to say	4	1.7
Aboriginal and/or Torres Strait Islander origin (***n*** = 232)		
Aboriginal	3	1.3
Not Aboriginal or Torres Strait Islander	226	97.4
Prefer not to say	3	1.3
Length of time since diagnosis (***n*** = 232)		
< 3 years ago	151	65.1
≥ 3 years or more	81	34.9
Ovarian cancer diagnosis sub-type (***n*** = 236)		
High grade serous	166	70.3
Low grade serous	23	9.7
Rare sub-type/other (i.e. clear cell, germ cell, carcinosarcoma)	24	10.2
Unsure/can’t remember	23	9.7
Healthcare service type (***n*** = 232)		
Public	91	39.2
Private	93	40.1
Both public and private	48	20.7

*Note: missing data were due to some survey respondents exiting the survey early. Any survey respondents who completed questions beyond the eligibility criteria were included in the final results

## Knowledge and perceptions on ovarian cancer clinical trials

Self-reported knowledge and importance level regarding clinical trials for ovarian cancer were assessed using two questions. Survey respondents rated these measures on a scale of 0 to 10, where 0 indicated no knowledge/importance and 10 indicated extremely knowledgeable/important. Most participants (33.1%, *n* = 88) reported a knowledge level of 5/10, with a mean response rating of 4.46 (SD = 2.34). Most (60.8%, *n* = 161) rated the importance of ovarian cancer clinical trials as extremely important at the highest rating of 10/10, with a mean response rating of 9.27 (SD = 1.17).

**Fig. 1:**
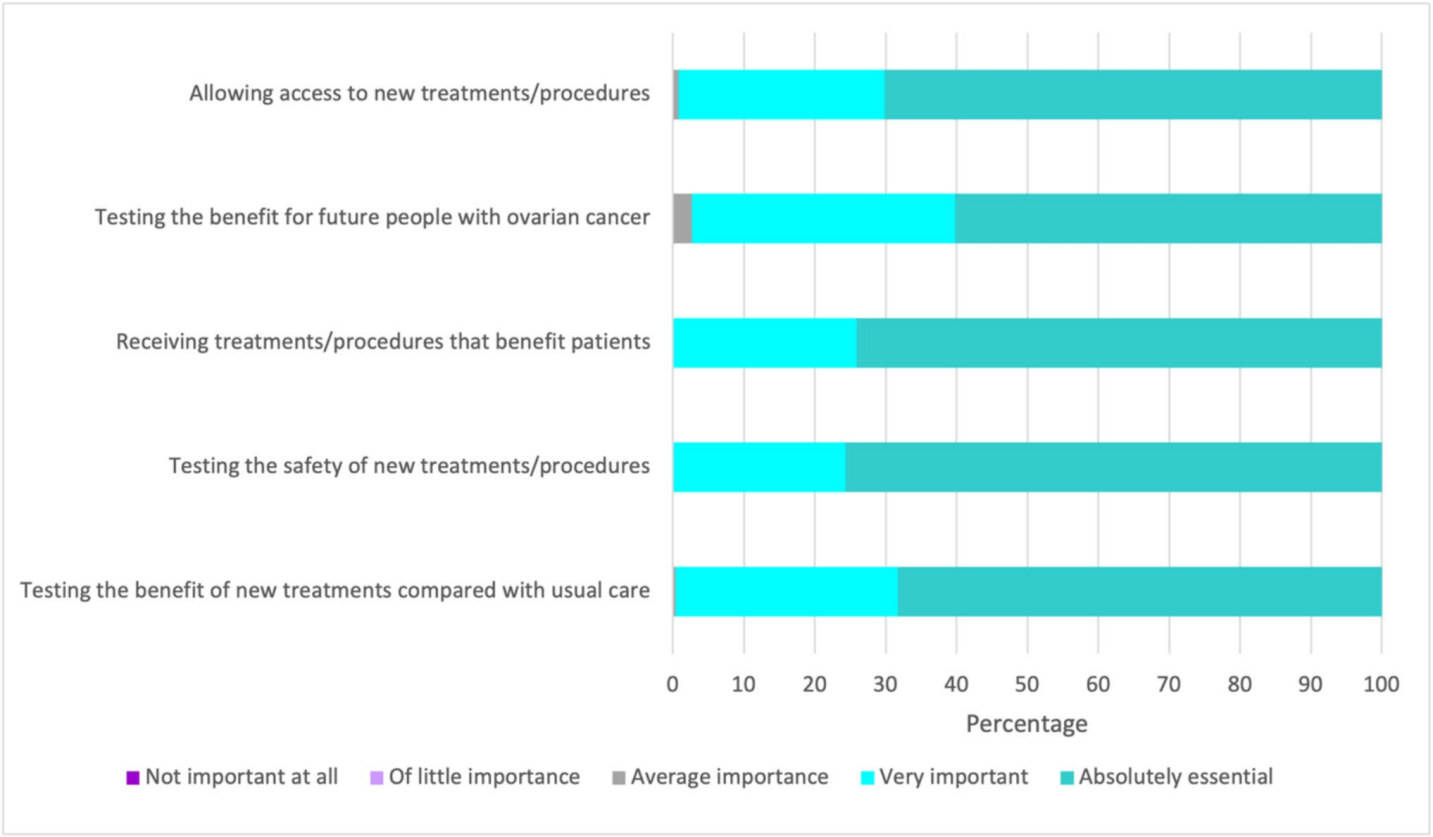
Perceptions on the role of clinical trials in ovarian cancer

Perceptions of importance on five statements outlining potential benefits of clinical trials for ovarian cancer were measured on a five-point scale from not important at all to absolutely essential. Most respondents rated all five statements as either absolutely essential or very important (see Fig. 1).

## Ovarian cancer clinical trials information access

Most respondents (56.2%, *n* = 145) had not received information on clinical trials from a health professional and an additional 6.6% (*n* = 17) couldn’t remember. Almost half (43.8%, *n* = 113) had looked for clinical trial information themselves (See Supplementary File 4 Table 1).

Of people (*n* = 112) who had looked for clinical trial information themselves, the four most frequently reported information sources were ‘Ovarian Cancer Australia website/social media’ (88.4%, *n* = 99); cancer doctor/oncologist/surgeon (84.8%, *n* = 95); Cancer Council website/social media (75%, *n* = 84); and Australia New Zealand Gynaecological Oncology Group website/social media (65.2%, *n* = 73).

Preferences for information formats, irrespective of whether respondents had previously looked for information themselves, were examined with participants (*n* = 255) selecting their level of preference on a 5-point scale. The five formats selected most frequently as either ‘definitely’ or ‘quite’ on the scale were email newsletter (81.2%, *n* = 207); discussion with a research professional (78.9%, *n *= 201); discussion with a health professional (78.5%, *n* = 200); a centralised online hub (76.9%, *n* = 196); and online written information (69.7%, *n* = 177). When asked to choose their most preferred format from the same list, email newsletter (33.5%, *n* = 85) was the most preferred, followed by discussion with a health professional (20.1%, *n* = 51) and a centralised online hub (16.5%, *n* = 42).

**Fig. 2:**
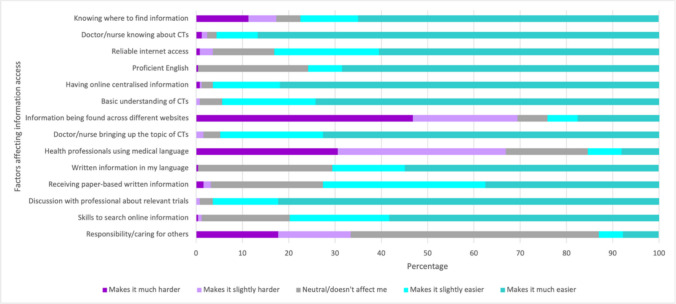
Extent to which factors affect access to information on clinical trials

Enablers and barriers to clinical trials information access were assessed with participants selecting the extent to which listed factors affect their access (see Fig. 2). Factors identified as making it easier to access information about clinical trials included: having a doctor or nurse who knows about clinical trials; talking to a person about clinical trials relevant to their cancer; and having one centralised place online to find ovarian cancer clinical trials information. Factors that made information access harder included: health professionals using medical language; clinical trials information being found across multiple websites; and being responsible or caring for others.

## Ovarian cancer clinical trials participation

Ovarian cancer clinical trial participation was confirmed by 26.1% (*n* = 64) of survey respondents and 7.3% (*n* = 18) were unsure of their participation. Regarding consideration of participating in a trial, 85.9% (*n* = 55) of those who had previously participated and 79.1% (*n* = 129) of those who hadn’t, responded that they would consider participating in a trial in the future.

**Fig. 3:**
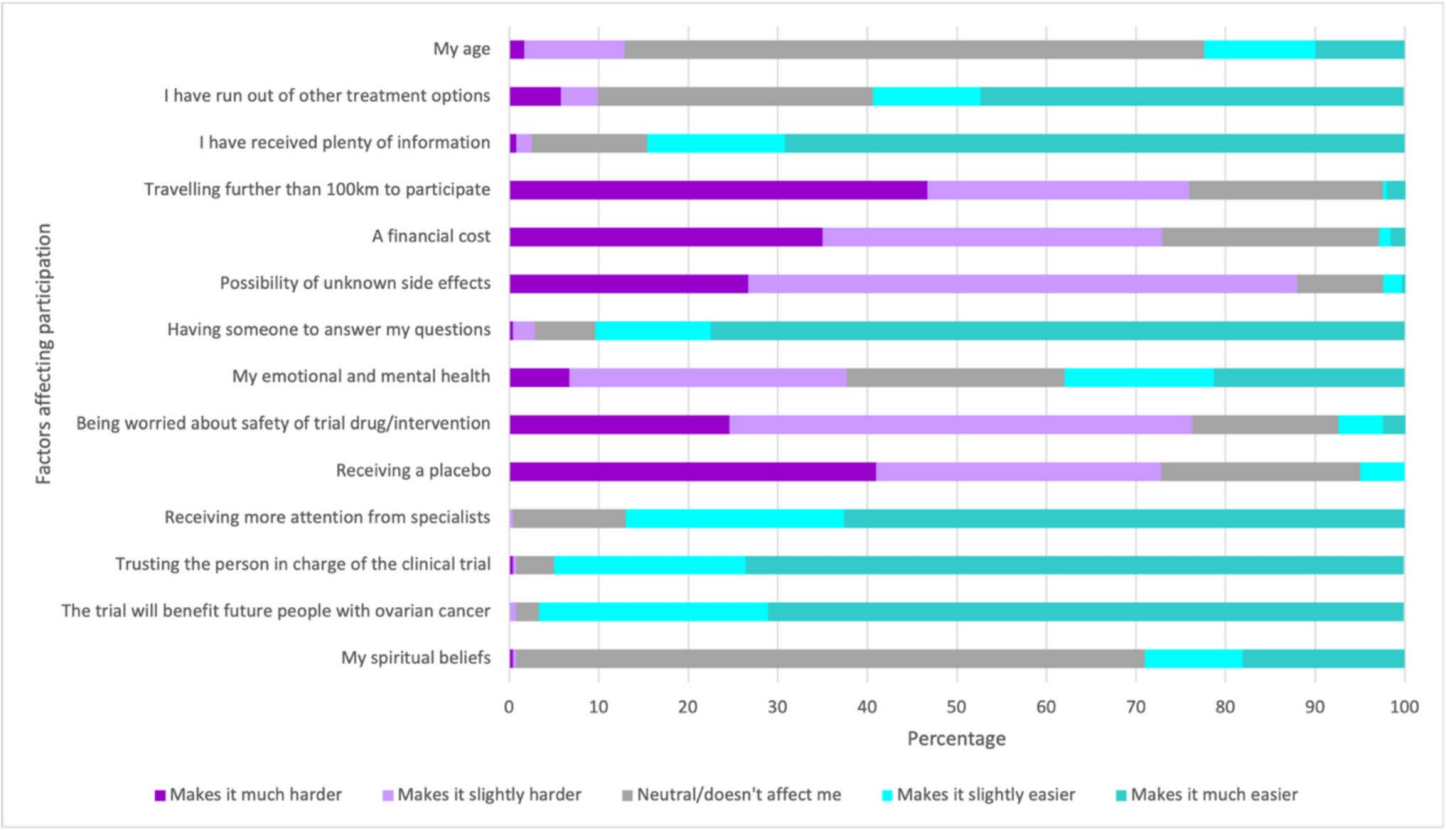
Extent by which factors affect participation in a trial

The most common factors to make it either slightly or much easier to participate in a clinical trial amongst survey respondents included the trial benefiting future people with ovarian cancer (96.6%, *n* = 230), trusting the person in charge of the trial (94.9%, *n* = 226), receiving more attention from specialists (87%, *n* = 207), and receiving plenty of information (84.6%, *n* = 203). Conversely, the most common factors that made it either slightly or much harder to participate in clinical trials were identified as the possibility of unknown side effects (88%, *n* = 211), being worried about the safety of the drug/intervention (76.3%, *n* = 183), and having to travel further than 100 km (75.9%, *n* = 182). See Fig. 3.

## Associations between participant characteristics and survey responses

There is a significant association between having received information on CTs from a health professional and length of time since diagnosis, which aligns with existing treatment limitations in later-stage or recurrent disease. Similarly, an association between length of time since diagnosis and trial participation, specifically people diagnosed more recently tended not to participate (Supplementary File 4 Table 2).

**Table 2:** Clinical trial participation

	Yes *n* (%)	No *n* (%)	Unsure *n* (%)
Have you participated in a clinical trial for ovarian cancer? (*n* = 245)	64 (26.1)	163 (66.5)	18 (7.3)
If yes, would you consider participating in another clinical trial for ovarian cancer? (*n* = 64)	55 (85.9)	1 (1.6)	8 (12.5)
If no, would you consider participating in a clinical trial for ovarian cancer? (*n* = 163)	129 (79.1)	3 (1.8)	31 (19.0)

People were less likely to have looked for information on clinical trials themselves if they were diagnosed up to 3 years ago or more likely to have looked for information if diagnosed with a high grade serous sub-type of ovarian cancer (Supplementary File 4 Table 2). These respondents were also more likely to have used ANZGOG, Cancer Council, Ovarian Cancer Australia, Ovarian Cancer Research Foundation, their treating health professionals, and other people with ovarian cancer as information sources (Supplementary File 4 Table 3). It should also be noted that another significant factor that influenced respondents’ participation in CTs included the need to travel distances over 100 km to do so, which was more prevalent in respondents aged 60 years or older (Supplementary File 4 Table 4).

**Table 3:** Qualitative analysis of open-ended question responses

Theme/subtheme	Definition	Example quotes
Theme 1: We need better solutions to help us find information and participate in clinical trials for ovarian cancer	Survey respondents explained that existing clinical trials information sources for ovarian cancer are not meeting their needs and better solutions are needed. They provided a range of suggestions to either improve existing clinical trial information sources or provided new ideas. Some of these ideas were introduced in the preceding survey content (i.e. a centralised online information hub and email newsletter) and others were additional	*One centralised hub with all the information, how to access, what's involved. An added bonus would be a health professional that is separate from your main team to discuss the trial with so to get an objective opinion.* (P78) *Having a reliable, up to date and well-maintained single portal for ovarian cancer clinical trials is really important. If that is unable to sustainably be supported into the future, the only other avenue for information is from your oncologist or cancer nurse.* (P263)
Subtheme: It’s difficult to find a trial for ovarian cancer	Survey respondents shared that it is difficult to find information about clinical trials or to participate in them for a range of reasons. These reasons were often shared in the context of their own experience	*It's really difficult. It's a specialist area but information is everywhere and fragmented. It's also hard to know which ones have succeeded in other countries yet not available in Australia. I wouldn't know where to start and that's the problem.* (P49) *Make it easier to access a trial. Eligibility criteria can be very strict, and women are missing out on trials that could help them.* (P276) *Chemo causes horrid brain fog. Finding info yourself can be really challenging. Even if info is found, it can be difficult to process. Having a medical professional talk through info, using plain language, is really important.* (P36)
Subtheme: Provide easy to understand information in multiple formats that can be discussed with health professionals	Survey respondents suggested that clinical trials information needs to be provided in language without medical jargon and in a variety of formats, both paper-based and digital. They described wanting to use this information to facilitate conversations with their treating team about clinical trials	*A regular newsletter, then you can read/digest at your own time. Some people don’t like support groups or talking too much about their situation.* (P325) *Making sure all information is accessible and written in easy English…. Not everyone can understand medical terminology.* (P165) *Getting the information in a medical setting—e.g. from an oncologist or Teal nurse—means it's less upsetting, as it's in the context of your overall health at the time of the appointment.* (P213) *Face to face discussion and easy to read and understand written information in the one place is always easier.* (P117)
Subtheme: Tell me what’s available and how to access trials including lifestyle interventions	Survey respondents suggested that they wanted to know more about what trials are available and how to access them. In particular, some respondents were interested in finding out more about clinical trials involving holistic or lifestyle interventions	*This survey has prompted me to question whether I could participate in trials related to exercise, diet and other lifestyle factors. I would be very keen to participate in any trials available to me.* (P85) *A newsletter with personal accounts included in a tactful but honest way would also be good for women unfamiliar with ovarian cancer.* (P171) *A central hub with all Australian clinical trials including outcomes designed for non-medical people in clear and easy to understand English. Also links to recent research papers published about clinical trials.* (P221)
Theme 2: Weighing up options: the decision to participate is affected by a variety of things	Survey participants shared experiences and opinions on factors they felt influenced their decision to participate in a clinical trial These ranged from considering the psychological impact of participating in a trial (including the potential for allocation of a placebo), travelling to the trial site, past experience of participating in trials and difficulty in finding information The influence of whether health professionals actively offered clinical trials information to them or not was also mentioned. Some respondents described examples where they felt they had missed out on supports and information because they were not offered by their treating team	*I participated in a clinical trial for ovarian cancer. Unfortunately I had to cease the trial due to bad side effects caused from the drugs used in the clinical trial. It made me quite sick for many months so I would be quite apprehensive participating again in a clinical trial.* (P38) *I had to travel from [regional area] to [capital city] monthly for two years. This became exhausting and expensive even with refunds. Trial availability in regional areas is essential.* (P100) *It is difficult to decide the best time to access a trial. Do you do it when current treatment seems to be keeping the disease stable, or do you try a new treatment? When is it too late to go on a trial…..disease too advanced. Timing is a big issue for me.* (P119) *At the moment the only way to access any information is if my Dr suggests something. I have looked and the only trials I can see are around drugs. I would happily participate in trials based on lifestyle. (*P125) *The frequent requirement for scans *etc. *is not necessarily reassuring. It is in one way if the results are encouraging but it also means more frequent 'scanxiety' and no chance to settle into a routine without anxiety over test/scan results.* (P53)
Theme 3: We want to help improve outcomes for women in the future	In the context of providing feedback on how to improve access to information and participation in clinical trials, survey respondents described that wanting to help improve outcomes for others with ovarian cancer in the future along with personal benefit motivated their desire to access information and participate in trials	*I need hope so l am willing to do any trial to help me live longer. I am praying for a miracle also if l can do a trial that will help others in the future that would be a good thing too.* (P95) *All patients should have access to hope to improve. Clinical trials could provide that hope. (*P79) *The experience of being diagnosed with OC* [ovarian cancer] *stage 4 made me very keen to help other women who discover they are on this path in future. To contribute by participating in a clinical trial would be an honour.* (P290)

## Suggestions for improvement

Open-ended responses on how to improve access to information and participation in clinical trials were answered by 96 participants. Three themes and three sub-themes emerged and are presented in Table 3 using a coding system to link quotations with the corresponding participant (p) and their anonymous survey identification number.

The first theme revealed that women need better solutions to help them find information and participate in clinical trials for ovarian cancer beyond existing resources. They explained difficulties faced in finding trials, for example, fragmentation of information across a range of sources, particular websites being hard to access or understand, the effect of treatment side effects such as cognitive disturbance or ‘brain fog’ and strict eligibility criteria limiting access to trials. Women suggested they want to be provided with easy-to-understand information in various formats, such as email newsletters, face-to-face discussions with health professionals and trusted websites. They also wanted to be informed about what clinical trials are available, including both trials involving drugs and lifestyle interventions.

In theme two, women described weighing up options when deciding to participate in a clinical trial. Factors affecting decisions included travel to trial sites, psychological impacts of trial participation, past experiences of trials, and difficulties finding information.

The third theme described how trial participation is affected by a desire to help improve outcomes for future women with ovarian cancer. Coupled with a sense of hope to improve access to better treatments and outcomes, women were altruistically motivated by an opportunity to improve both their own prognosis and that of others.

## Discussion

This study provides a national snapshot of clinical trial awareness, information access, and participation among Australians with ovarian cancer. Despite high perceived importance of clinical trials, self-rated knowledge and participation were modest. Over half of respondents had not received information about clinical trials from health professionals, and a substantial proportion sought information independently. Participants expressed a strong preference for centralised, accessible information delivered through trusted formats, particularly email newsletters, health professional discussions, and online hubs. Several demographic characteristics were associated with information behaviours and participation drivers, suggesting clinicians may benefit from anticipating and tailoring communication to the needs of particular patient groups.

### Barriers and enablers to clinical trial awareness, information, and participation

Barriers to clinical trial information access, such as fragmented sources, complex medical language, and competing caregiving responsibilities, were prominent in this cohort. Although the effectiveness of interventions to address such barriers remains limited [34], there is growing recognition that enabling informed decision-making through clear, person-centred communication is fundamental to delivering cancer care that matters most to patients [35]. A multifaceted approach is required to improve awareness and participation in trials, alongside supportive care services for participants with special needs and in ways that are culturally adaptive [36].

Despite existing online registries and professional databases, our results confirm available resources do not adequately meet the informational needs of this cohort. Issues with content accessibility, readability, and navigation continue to impede patient engagement. International reviews of trial recruitment challenges report similar themes: poor understanding of trial concepts, rushed or unclear communications, and patients feeling ill-equipped to weigh the risks of benefits [11, 37, 38]. Similar to the perspectives of Australians with ovarian cancer [10], cancer patients use online sources to seek clarification of confusing terminology and describe the need for tailored information [38]. Evaluating the risks and benefits of trial participation is identified globally as a patient consideration, along with the hope for both a personal benefit and to help others [11, 37]. These findings align closely with the views of Australians in this study, who highlighted the desire for tailored, timely, and credible information. Suggested solutions include simplified brochures, online tools, and clinician-guided discussions [37, 38].

Although online delivery and clinician-guided discussions were preferred modalities of our study participants, these pose their own challenges. International studies show that clinical trial content often exceeds recommended reading levels [39, 40], with similar findings reported for gynaecological cancer resources in Australia [41]. Accessibility issues underscore the need for co-designed resources that are linguistically appropriate, culturally inclusive, and adaptable to health literacy levels.

### Practical and policy implications

In response to the study findings, development of a dedicated online clinical trials resource tailored for Australians with ovarian cancer is underway. This resource will be developed in partnership with health communication experts to ensure plain-language content and include translation tools to meet the needs of culturally and linguistically diverse users. In addition to written content, videos, webinars, and interactive elements are recommended to accommodate varied learning preferences. Ongoing co-design with people with lived experience, guided by NSQHS standards [42], will enhance relevance, acceptability, and impact.

Beyond digital resources, continued collaboration with sector partners is essential to evaluate and iterate interventions. Supportive care models, such as the OCA Teal Support Program, can play a crucial role in trial navigation and information access. Sector-wide advocacy to increase trial availability and reduce geographic barriers is also critical, especially in a country as large and diverse as Australia. Sustained investment is needed to build equitable systems that support participation in clinical research, preserve quality of life, and improve survival outcomes.

This project adopted an approach to building a comprehensive clinical trials information resource underpinned by evidence collected from people with lived experience. The identified enablers and barriers to this Australian population were foundational to ensuring resource content was guided by their needs. It serves as an example of how similar international resources may be developed to be contextually relevant to their intended audience. Future evaluation to measure resource use and efficacy to improve awareness and access to clinical trials information is recommended along with better understanding how histological sub-type, types of available trials, and timing of information access relative to diagnosis affect clinical trial awareness and participation.

### Strengths and limitations

The design and conduct of this study offer several notable strengths. This is the first study to collectively investigate clinical trials awareness, information access and participation amongst Australians with ovarian cancer, addressing a significant gap in research literature. Participants represented a large, national sample of individuals with recent ovarian cancer experience. Although modest, the sample included representation of cultural diversity including Aboriginal Australians. These enhance relevance to a broad Australian context, though readers are encouraged to consider whether the demographic profile of participants reflects their populations when applying the results. Using a mixed methods design enabled a richer exploration of experiences and perspectives than a purely quantitative survey approach. Integration of both survey metrics and qualitative insights allowed for a more nuanced understanding of the factors influencing clinical trial engagement. The survey instrument was developed through a collaborative, stakeholder informed process strengthening its content validity and relevance.

Several limitations should be acknowledged. The study population was restricted to people diagnosed with or experiencing recurrence of ovarian cancer within 5 years, to minimise recall bias. However, this may have inadvertently excluded long-term survivors or those with earlier exposure to clinical trials, potentially underestimating cumulative participation. Recruitment primarily through OCA networks may have introduced selection bias, particularly in participants’ familiarity with existing resources promoted by OCA. Due to the nature of recruitment through multiple modes such as email distribution, social media posts and a webpage, a response rate was unable to be calculated. In addition, while a range of information sources and barriers were assessed through predefined survey items, these may not have captured the full breadth of experiences. As with all self-reported data, the potential for response bias should be considered.

## Conclusions

This national study highlights critical gaps in clinical trials knowledge, information access and participation among Australians with ovarian cancer. Driven by the voices of people with lived experience, it addresses a significant gap in research by exploring their perspectives, preferences, and unmet needs. The findings reveal modest overall knowledge and a clear desire for easy-to-understand information, particularly when delivered by health professionals and through accessible digital formats.

Participants’ insights point to a tangible opportunity to optimise equity in information delivery. Informed by these results, the development of a publicly available, centralised online resource tailored to the ovarian cancer context is underway. This initiative adopts a co-design approach to ensure the resource meets the diverse needs of its intended audience. Continued sector collaboration, inclusive communication strategies, and investment in supportive care models will be essential to empower all Australians with ovarian cancer to make informed choices about clinical trial participation.

## Supplementary Information

Below is the link to the electronic supplementary material.

ESM 1 (PDF 265 KB)

ESM 2 (PDF 274 KB)

ESM 3 (PDF 428 KB)

ESM 4 (PDF 134 KB)

## Data Availability

The data are not publicly available due to privacy.

## References

[1] Chung KC, Muthutantri A, Goldsmith GG, Watts MR, Brown AE, Patrick DL (2024) Symptom impact and health-related quality of life (HRQoL) assessment by cancer stage: a narrative literature review. BMC Cancer 24:884–823 39039461 10.1186/s12885-024-12612-z PMC11265440

[2] Zahren C, Harvey S, Weekes L, Bradshaw C, Butala R, Andrews J, O’Callaghan S (2021) Clinical trials site recruitment optimisation: guidance from clinical trials: impact and quality. Clinical trials (London, England) 18:594–605 34041937 10.1177/17407745211015924 PMC8479150

[3] Mosconi P, Roberto A, Cerana N, Colombo N, Didier F, D’Incalci M, Lorusso D, Peccatori FA, Artioli G, Cavanna L, Ceccherini R, Cirigliano G, Comerci G, Cormio G, Crippa A, Farolfi A, Febbraro A, Giardina D, Greggi S, Lalle M, Lapresa M, Marzola M, Merisio C, Mosconi AM, Peiretti M, Ricci G, Ronzino G, Scambia G, Scollo P, Sina F, Stella GC, Tomao F, Vici P, Zola P, Network of C, Participants (2022) Knowledge and attitudes towards clinical trials among women with ovarian cancer: results of the ACTO study. Journal of Ovarian Research 15: 45 10.1186/s13048-022-00970-w PMC9010065 35422000

[4] Roczo D, Alford V, Trainer A, DeFazio A, Pearn A, Delaney C, Cotter M, Hegarty S (2024) Self-reported awareness of genetic testing, the impact of family history, and access to clinical trials for people diagnosed with ovarian cancer in Australia. Gynecol Oncol Rep 54:101427 38989471 10.1016/j.gore.2024.101427 PMC11233906

[5] Bazarbashi S, Hassan A, Eldin AM, Soudy H, Hussain F (2015) Awareness and perceptions of clinical trials in cancer patients and their families in Saudi Arabia. J Cancer Educ 30:655–659 25663358 10.1007/s13187-015-0797-0

[6] Engebretson A, Matrisian L, Thompson C (2016) Patient and caregiver awareness of pancreatic cancer treatments and clinical trials. J Gastrointest Oncol 7:228–233 27034790 10.3978/j.issn.2078-6891.2015.102 PMC4783733

[7] Lim Y, Lim JM, Jeong WJ, Lee KH, Keam B, Kim TY, Kim TM, Han SW, Oh DY, Kim DW, Kim TY, Heo DS, Bang YJ, Im SA (2017) Korean cancer patients’ awareness of clinical trials, perceptions on the benefit and willingness to participate. Cancer Res Treat 49:1033–1043 28392549 10.4143/crt.2016.413 PMC5654169

[8] Staniszewska A, Lubiejewska A, Czerw A, Dabrowska-Bender M, Duda-Zalewska A, Olejniczak D, Juszczyk G, Bujalska-Zadrozny M (2018) Awareness and attitudes towards clinical trials among Polish oncological patients who had never participated in a clinical trial. Advances in clinical and experimental medicine: official organ Wroclaw Medical University 27:525–529 29616747 10.17219/acem/68762

[9] Wigginton B, Reeves MM, DiSipio T (2023) Exploring motivations for participating in research among Australian women with advanced gynaecological cancer: a qualitative study. Support Care Cancer 31:511 37552313 10.1007/s00520-023-07979-x PMC10409726

[10] Williams N, Russell H, Bradhurst B (2025) Exploring clinical trials awareness, information access and participation amongst Australians with ovarian cancer: a qualitative study. Support Care Cancer 33:176 39934363 10.1007/s00520-025-09221-2 PMC11814028

[11] Houghton C, Dowling M, Meskell P, Hunter A, Gardner H, Conway A, Treweek S, Sutcliffe K, Noyes J, Devane D, Nicholas JR, Biesty LM (2020) Factors that impact on recruitment to randomised trials in health care: a qualitative evidence synthesis. The Cochrane database of systematic reviews 10: Mr000045 10.1002/14651858.MR000045.pub2 PMC8078544 33026107

[12] Unger JM, Vaidya R, Hershman DL, Minasian LM, Fleury ME (2019) Systematic review and meta-analysis of the magnitude of structural, clinical, and physician and patient barriers to cancer clinical trial participation. JNCI J Natl Cancer Inst 111:245–255 30856272 10.1093/jnci/djy221 PMC6410951

[13] Ford J, Howerton M, Bolen S, Gary-Webb T, Lai G, Tilburt J, Gibbons M, Baffi C, Wilson R, Feuerstein C, Tanpitukpongse P, Powe N, Bass E (2005) Knowledge and access to information on recruitment of underrepresented populations to cancer clinical trials. Evid Rep Technol Assess (Summary):1–11. 10.1037/e439572005-001 10.1037/e439572005-001 PMC4781635 15989377

[14] Omico (2025) Cancer Screening Program - CaSP. https://www.omico.com.au/our-programs/cancer-screening-program-casp/. Accessed 11 Mar 2025

[15] Fetters MD, Curry LA, Creswell JW (2013) Achieving integration in mixed methods designs-principles and practices. Health Serv Res 48:2134–2156 24279835 10.1111/1475-6773.12117 PMC4097839

[16] Kesmodel US (2018) Cross-sectional studies – what are they good for? Acta Obstet Gynecol Scand 97:388–393 29453895 10.1111/aogs.13331

[17] Allemang B, Sitter K, Dimitropoulos G (2022) Pragmatism as a paradigm for patient-oriented research. Health Expect 25:38–47 34748689 10.1111/hex.13384 PMC8849373

[18] Vandenbroucke JP, von Elm E, Altman DG, Gotzsche PC, Mulrow CD, Pocock SJ, Poole C, Schlesselman JJ, Egger M (2007) Strengthening the reporting of observational studies in epidemiology (STROBE): explanation and elaboration. Epidemiology 18:805–835 18049195 10.1097/EDE.0b013e3181577511

[19] Eysenbach G (2004) Improving the quality of web surveys: the checklist for reporting results of internet E-surveys (CHERRIES). J Med Internet Res 6:e34 15471760 10.2196/jmir.6.3.e34 PMC1550605

[20] National Cancer Institute (2024) Ovarian epithelial, fallopian tube, and primary peritoneal cancer treatment (PDQ®)–Health Professional Version. https://www.cancer.gov/types/ovarian/hp/ovarian-epithelial-treatment-pdq. Accessed 12 Mar 2025 26389443

[21] Cancer Australia (2025) Ovarian cancer statistics in Australia. https://www.canceraustralia.gov.au/cancer-types/ovarian-cancer/statistics. Accessed 5 Mar 2025

[22] Leon RJ, Lapkin S, Fields L, Moroney T (2022) Developing a self-administered questionnaire: methods and considerations. Nurse Res 30:36–45 36043328 10.7748/nr.2022.e1848

[23] Chi J, Pian W, Zhang S (2020) Consumer health information needs: a systematic review of instrument development. Inf Process Manag 57:102376

[24] Lees C (2011) Measuring the patient experience. Nurse Res 19:25–28 22128584 10.7748/nr2011.10.19.1.25.c8768

[25] Östlund U, Kidd L, Wengström Y, Rowa-Dewar N (2011) Combining qualitative and quantitative research within mixed method research designs: a methodological review. Int J Nurs Stud 48:369–383 21084086 10.1016/j.ijnurstu.2010.10.005 PMC7094322

[26] Polit DF, Beck CT, Owen SV (2007) Is the CVI an acceptable indicator of content validity? Appraisal and recommendations. Res Nurs Health 30:459–467 17654487 10.1002/nur.20199

[27] Harris PA, Taylor R, Thielke R, Payne J, Gonzalez N, Conde JG (2009) Research electronic data capture (REDCap)—a metadata-driven methodology and workflow process for providing translational research informatics support. J Biomed Inform 42:377–381 18929686 10.1016/j.jbi.2008.08.010 PMC2700030

[28] IBM Corp (2023) IBM SPSS statistics for windows. Version 29.0. IBM Corp, Armonk.

[29] Hsieh H-F, Shannon SE (2005) Three approaches to qualitative content analysis. Qual Health Res 15:1277–1288 16204405 10.1177/1049732305276687

[30] Erlingsson C, Brysiewicz P (2017) A hands-on guide to doing content analysis. Afr J Emerg Med 7:93–99 30456117 10.1016/j.afjem.2017.08.001 PMC6234169

[31] The National Health and Medical Research Council [NHMRC], The Australian Research Council, Universities Australia (2018) The Australian code for the responsible conduct of research. https://www.nhmrc.gov.au/about-us/publications/australian-code-responsible-conduct-research-2018. Accessed 14 May 2024

[32] The National Health and Medical Research Council [NHMRC], The Australian Research Council, Universities Australia (2023) National Statement on Ethical Conduct in Human Research 2023. https://www.nhmrc.gov.au/about-us/publications/national-statement-ethical-conduct-human-research-2023. Accessed 14 May 2024

[33] World Medical Association (2024) WMA Declaration of Helsinki – ethical principles for medical research involving human participants. https://www.wma.net/policies-post/wma-declaration-of-helsinki/. Accessed 14 May 2024 10.1001/jama.2024.21972 39425955

[34] Michaels M, Weiss ES, Sae-Hau M, Illei D, Lilly B, Szumita L, Connell B, Lee M, Cooks E, McPheeters M (2024) Strategies for increasing accrual in cancer clinical trials: what is the evidence? Cancer Med 13:e7298 38770644 10.1002/cam4.7298 PMC11106681

[35] Booth CM, Sengar M, Goodman A, Wilson B, Aggarwal A, Berry S, Collingridge D, Denburg A, Eisenhauer EA, Ginsburg O, Goldstein D, Gunasekera S, Hammad N, Honda K, Jackson C, Karikios D, Knopf K, Koven R, Marini BL, Maskens D, Moraes FY, Mohyuddin GR, Poudyal BS, Pramesh CS, Roitberg F, Rubagumya F, Schott S, Sirohi B, Soto-Perez-de-Celis E, Sullivan R, Tannock IF, Trapani D, Tregear M, van der Graaf W, Vanderpuye V, Gyawali B (2023) Common sense oncology: outcomes that matter. Lancet Oncol 24:833–835 37467768 10.1016/S1470-2045(23)00319-4

[36] Chen J, Lu Y, Kummar S (2023) Increasing patient participation in oncology clinical trials. Cancer Med 12:2219–2226 36043431 10.1002/cam4.5150 PMC9939168

[37] Chichua M, Mazzoni D, Marzorati C, Pravettoni G (2025) The journey of patients in cancer clinical trials: a qualitative meta-synthesis on experiences and perspectives. Patient Educ Couns 130:108469 39426006 10.1016/j.pec.2024.108469

[38] Viljoen B, Chambers SK, Dunn J, Ralph N, March S (2020) Deciding to enrol in a cancer trial: a systematic review of qualitative studies deciding to enrol in a cancer trial: a systematic review of qualitative studies. J Multidiscip Healthc 13:1257–1281 33149597 10.2147/JMDH.S266281 PMC7603415

[39] Friedman DB, Kim S-H, Tanner A, Bergeron CD, Foster C, General K (2014) How are we communicating about clinical trials?: an assessment of the content and readability of recruitment resources. Contemp Clin Trials 38:275–283 24836075 10.1016/j.cct.2014.05.004

[40] Hillyer GC, Beauchemin M, Basch CH, Hillyer GC, Beauchemin M, Garcia P, Kelsen M, Brogan FL, Schwartz GK, Basch CH (2020) Readability of cancer clinical trials websites. Cancer Control 27:107327481990112 10.1177/1073274819901125 PMC6984426 31973569

[41] DiSipio T, Scholte C, Diaz A (2024) Evaluation of online text-based information resources of gynaecological cancer symptoms. Cancer Med 13:e7167 38676385 10.1002/cam4.7167 PMC11053368

[42] Australian Commission on Safety and Quality in Health Care [ACSQHC] (2021) National safety and quality health service standards (second edition). https://www.safetyandquality.gov.au/publications-and-resources/resource-library/national-safety-and-quality-health-service-standards-second-edition. Accessed 22 Apr 2025

