# Supplementary material for: Listening to Australians with ovarian cancer: a cross-sectional survey investigating clinical trials awareness, information access and participation
Source: Support Care Cancer. 2026 Mar 22;34(4):350. doi: 10.1007/s00520-026-10586-1 (PMC13005823; doi:10.1007/s00520-026-10586-1)
Supplement: Supplementary file 2 — (PDF 274 KB) [file 520_2026_10586_MOESM2_ESM.pdf]

SUPPLEMENTARY FILE 2

SURVEY TOOL

Title:

Listening to Australians with ovarian cancer: a cross-sectional survey investigating clinical trials awareness, information access and participation

Journal

Supportive Care in Cancer

Authors:

Natalie Williams; Yeh Chen Lee; Hayley Russell; John Andrews; Won Sun Chen; Bridget Bradhurst

Corresponding author: Natalie Williams, Ovarian Cancer Australia; Curtin University
natalie.f.williams@curtin.edu.au

Clinical Trials Awareness, Information Access and Participation (CT-AIP) Survey Tool

Consent declaration:

I have read the Participant Information Form for this research project and give consent to participate.

☐Yes

☐No (link to message of ineligibility)

Eligibility Criteria:

For the purposes of this survey the following definitions apply:

Ovarian cancer is a disease where cells in the ovary develop into a malignant tumour. The term ‘ovarian cancer’ also includes tumours occurring in the fallopian tube or peritoneal cavity due to their similarities. Cancer Australia (2023)

Have you received a diagnosis or recurrence of ovarian, fallopian tube or peritoneal cancer within the past five years?

☐Yes
☐No (link to message of ineligibility)

What is your age? (link to message of ineligibility if 17 or less)

______

Are you able to read and write in English?

☐Yes
☐No (branch to next question)

Branch from previous question - Do you have someone who can help you to interpret this survey into your own language?

☐Yes
☐No (link to message of ineligibility)

Do you live in Australia?

☐Yes
☐No (link to message of ineligibility)

Knowledge and Opinion of Clinical Trials

How do you rate your level of knowledge on the topic of clinical trials? (i.e. understanding what they are and how they are carried out)

0= I have no knowledge at all 5= I have moderate knowledge 10 = I am extremely knowledgeable

1 2 3 4 5 6 7 8 9 10

(Slider from 0 – 10)

How do you rate the importance of clinical trials in ovarian cancer care?

0 = They are not important at all = The are moderately important 10 = They are absolutely essential

1 2 3 4 5 6 7 8 9 10

(Slider from 0 – 10)

Note to Participant:

Clinical Trials Definition:

Clinical trials are a way to test either treatments (such as drugs), or interventions (such as giving a standard drug at different times or testing a lifestyle intervention like an exercise program). Researchers and scientists use them to work out which treatments or interventions work best so that people with a health condition can receive the best available care.

When thinking about the use of clinical trials in ovarian cancer care, how important are the following, in your opinion:

Testing if a new treatment/procedure works better than the usual treatment offered to patients

☐Not important at all
☐Of little importance
☐Average importance
☐Very important
☐Absolutely essential

Testing if a new treatment/procedure is safe and won't cause harm

☐Not important at all
☐Of little importance
☐Average importance
☐Very important
☐Absolutely essential

Receiving a treatment/procedure as part of a clinical trial that may benefit patients, for example helping patients to live longer or have a better quality of life

☐Not important at all
☐Of little importance
☐Average importance
☐Very important
☐Absolutely essential

Testing the impact of a new treatment/procedure may benefit future people with ovarian cancer even if it doesn't benefit the person in the trial

☐Not important at all
☐Of little importance
☐Average importance
☐Very important
☐Absolutely essential

Clinical trials allow access to a new treatment/procedure that can't be accessed any other way

☐Not important at all
☐Of little importance
☐Average importance
☐Very important
☐Absolutely essential

Finding Out About Clinical Trials

Have you received information on clinical trials for ovarian cancer from a health professional?

☐Yes
☐No
☐I can't remember

Have you looked for information on clinical trials for ovarian cancer yourself?

☐Yes (Branch to questions below)
☐No
☐I can't remember

Branch - If yes:

How often have you accessed the following places/people for information on clinical trials?

Australia New Zealand Gynaecological Oncology Group (ANZGOG) website/social media

☐Never
☐Once
☐A few times (2-3 times)
☐Frequently (4 or more times)
☐I can't remember

Australian and New Zealand Clinical Trials Registry (ANZCTR) website

☐Never
☐Once
☐A few times (2-3 times)
☐Frequently (4 or more times)
☐I can't remember

Cancer Council website/social media

☐Never
☐Once
☐A few times (2-3 times)
☐Frequently (4 or more times)
☐I can't remember

Ovarian Cancer Australia website/social media

☐Never
☐Once
☐A few times (2-3 times)
☐Frequently (4 or more times)
☐I can't remember

Ovarian Cancer Research Foundation website/social media

☐Never

☐Once
☐A few times (2-3 times)
☐Frequently (4 or more times)
☐I can’t remember

My cancer doctor/oncologist/surgeon

☐Never
☐Once
☐A few times (2-3 times)
☐Frequently (4 or more times)
☐I can’t remember

My GP (general practitioner doctor)

☐Never
☐Once
☐A few times (2-3 times)
☐Frequently (4 or more times)
☐I can’t remember

A cancer nurse at my treatment centre/hospital (not Teal Support/OCA nurse)

☐Never
☐Once
☐A few times (2-3 times)
☐Frequently (4 or more times)
☐I can’t remember

Ovarian Cancer Australia nurse (including Teal Support Nurse)

☐Never
☐Once
☐A few times (2-3 times)
☐Frequently (4 or more times)
☐I can’t remember

Other people with ovarian cancer including online support groups

☐Never
☐Once
☐A few times (2-3 times)
☐Frequently (4 or more times)
☐I can’t remember

Alternative health providers (i.e. Naturopath)

☐Never

☐Once
☐A few times (2-3 times)
☐Frequently (4 or more times)
☐I can’t remember

Family, friends, or work colleagues

☐Never
☐Once
☐A few times (2-3 times)
☐Frequently (4 or more times)
☐I can’t remember

Please list other clinical trials information sources have you accessed? (including international) If none, leave blank:

____

Would you like to access or receive information about clinical trials relevant to your current health in the following ways?

An email newsletter

☐No/not at all
☐Slightly
☐Moderately
☐Quite
☐Definitely

A text message on my phone

☐No/not at all
☐Slightly
☐Moderately
☐Quite
☐Definitely

Posts on social media channels such Facebook and Instagram

☐No/not at all
☐Slightly
☐Moderately
☐Quite
☐Definitely

A discussion with a health professional

☐No/not at all
☐Slightly
☐Moderately
☐Quite
☐Definitely

A discussion with a research professional (e.g. Research nurse or trial coordinator) who works in clinical trials

☐No/not at all
☐Slightly
☐Moderately
☐Quite
☐Definitely

Written information on a website

☐No/not at all
☐Slightly
☐Moderately
☐Quite
☐Definitely

A centralised online hub (an online collection of information about ovarian cancer trials)

☐No/not at all
☐Slightly
☐Moderately
☐Quite
☐Definitely

Written information as a paper-based brochure/flyer

☐No/not at all
☐Slightly
☐Moderately
☐Quite
☐Definitely

Online videos to watch such as via YouTube

☐No/not at all
☐Slightly
☐Moderately
☐Quite
☐Definitely

Online learning resources (i.e. webinars)

☐No/not at all
☐Slightly
☐Moderately
☐Quite
☐Definitely

Please list other ways would you like to access or receive information about clinical trials (if none, leave blank): ______________________________________

Please select your most preferred way of receiving or accessing information about clinical trials? (select one from list below)

☐An email newsletter

☐A text message on my phone

☐Posts on social media channels such Facebook and Instagram

☐A discussion with a health professional

☐A discussion with a research professional who works in clinical trials

☐Written information on a website

☐A centralised online hub (an online collection of information about ovarian cancer trials)

☐Written information as a paper-based brochure/flyer

☐Online videos to watch such as via YouTube

☐Online learning resources (i.e. webinars)

☐Other ________________________________ (branch to question below)

Branch from “Other” – What is your most preferred way of receiving or accessing information about clinical trials?

____

To what extent might the following things make it harder or easier for you to access information about ovarian cancer clinical trials:

(Please note this list includes things that might make it harder or easier for you to access information)

5 point Likert: (Makes it much harder| Makes it slightly harder| Neutral/doesn't affect me| Makes it slightly easier| Makes it much easier)

Knowing where to find information about clinical trials

☐Makes it much harder
☐Makes it slightly harder
☐Neutral/doesn't affect me
☐Makes it slightly easier
☐Makes it much easier

Having a doctor or nurse who knows about clinical trials for ovarian cancer

☐Makes it much harder
☐Makes it slightly harder
☐Neutral/doesn't affect me
☐Makes it slightly easier
☐Makes it much easier

Reliable internet access

☐Makes it much harder
☐Makes it slightly harder
☐Neutral/doesn't affect me
☐Makes it slightly easier
☐Makes it much easier

Speaking/understanding English well

☐Makes it much harder
☐Makes it slightly harder
☐Neutral/doesn't affect me
☐Makes it slightly easier
☐Makes it much easier

Having one centralised place online to find information on clinical trials for ovarian cancer

☐Makes it much harder
☐Makes it slightly harder
☐Neutral/doesn't affect me
☐Makes it slightly easier
☐Makes it much easier

Having a basic understanding of what clinical trials are

☐Makes it much harder
☐Makes it slightly harder
☐Neutral/doesn’t affect me
☐Makes it slightly easier
☐Makes it much easier

Information on clinical trials for ovarian cancer being found separately across many different websites

☐Makes it much harder
☐Makes it slightly harder
☐Neutral/doesn’t affect me
☐Makes it slightly easier
☐Makes it much easier

Having a doctor or nurse bring up the topic of clinical trials

☐Makes it much harder
☐Makes it slightly harder
☐Neutral/doesn’t affect me
☐Makes it slightly easier
☐Makes it much easier

Health professionals using medical language when discussing clinical trials

☐Makes it much harder
☐Makes it slightly harder
☐Neutral/doesn’t affect me
☐Makes it slightly easier
☐Makes it much easier

Having written information about clinical trials in my preferred language

☐Makes it much harder
☐Makes it slightly harder
☐Neutral/doesn’t affect me
☐Makes it slightly easier
☐Makes it much easier

Being given written information in paper-based formats (i.e. brochure, flyer)

☐Makes it much harder
☐Makes it slightly harder
☐Neutral/doesn’t affect me

☐Makes it slightly easier
☐Makes it much easier

Talking to a person about trials that are relevant to my ovarian cancer (such as a doctor/nurse or a research professional)

☐Makes it much harder
☐Makes it slightly harder
☐Neutral/doesn’t affect me
☐Makes it slightly easier
☐Makes it much easier

Having the computer skills to look for online information

☐Makes it much harder
☐Makes it slightly harder
☐Neutral/doesn’t affect me
☐Makes it slightly easier
☐Makes it much easier

Being responsible or caring for others e.g. children/parents

☐Makes it much harder
☐Makes it slightly harder
☐Neutral/doesn’t affect me
☐Makes it slightly easier
☐Makes it much easier

Things that affect clinical trials participation

Reminder: Clinical trials can test either treatments (such as drugs), or interventions (such as giving a standard drug at different times, or testing a lifestyle intervention like an exercise program).

Have you participated in a clinical trial for ovarian cancer?

☐Yes (Branch to “if yes” questions below)
☐No (Branch to “if no” question below)
☐Unsure

If yes, would you consider participating in another clinical trial for ovarian cancer?

☐Yes
☐No
☐Unsure

If yes, what type of trial have you participated in? (select all that apply)

☐Clinical trial testing a drug
☐Clinical trial testing an intervention (such as testing a lifestyle intervention like an exercise program, or a new way of giving a drug)
☐Other (Branch to question below)
☐I can’t remember/unsure

Branch from “Other research” – What other type of trial have you participated in?

____

If no, would you consider participating in a clinical trial for ovarian cancer?

☐Yes
☐No
☐Unsure

If there was a clinical trial available to you, to what extent might the following things make it harder or easier for you to join or decide to join the trial:

My age

☐Makes it much harder
☐Makes it slightly harder
☐Neutral/doesn't affect me
☐Makes it slightly easier
☐Makes it much easier

I have run out of other treatment options

☐Makes it much harder
☐Makes it slightly harder
☐Neutral/doesn't affect me
☐Makes it slightly easier
☐Makes it much easier

I have received plenty of information about the trial

☐Makes it much harder
☐Makes it slightly harder
☐Neutral/doesn't affect me
☐Makes it slightly easier
☐Makes it much easier

I would have to travel further than 100km to participate in the clinical trial

☐Makes it much harder
☐Makes it slightly harder
☐Neutral/doesn't affect me
☐Makes it slightly easier
☐Makes it much easier

There would be a financial cost to me (i.e. needing money to travel or stay overnight)

☐Makes it much harder
☐Makes it slightly harder
☐Neutral/doesn't affect me
☐Makes it slightly easier
☐Makes it much easier

The possibility of experiencing unknown side effects

☐Makes it much harder
☐Makes it slightly harder

☐Neutral/doesn't affect me
☐Makes it slightly easier
☐Makes it much easier

Having someone to answer my questions

☐Makes it much harder
☐Makes it slightly harder
☐Neutral/doesn't affect me
☐Makes it slightly easier
☐Makes it much easier

My emotional and mental health, and well-being

☐Makes it much harder
☐Makes it slightly harder
☐Neutral/doesn't affect me
☐Makes it slightly easier
☐Makes it much easier

I was worried about the safety of the drug or intervention being tested

☐Makes it much harder
☐Makes it slightly harder
☐Neutral/doesn't affect me
☐Makes it slightly easier
☐Makes it much easier

During the trial I might receive a placebo (e.g. sugar pill or fake procedure) or standard treatment instead of an active drug or new treatment plan

☐Makes it much harder
☐Makes it slightly harder
☐Neutral/doesn't affect me
☐Makes it slightly easier
☐Makes it much easier

I would receive more attention from specialist cancer doctors and other health professionals

☐Makes it much harder
☐Makes it slightly harder
☐Neutral/doesn't affect me
☐Makes it slightly easier
☐Makes it much easier

I trust the person who is in charge of the clinical trial and their recommendation

☐Makes it much harder
☐Makes it slightly harder
☐Neutral/doesn’t affect me
☐Makes it slightly easier
☐Makes it much easier

This trial will benefit people with ovarian cancer in the future

☐Makes it much harder
☐Makes it slightly harder
☐Neutral/doesn’t affect me
☐Makes it slightly easier
☐Makes it much easier

My spiritual beliefs

☐Makes it much harder
☐Makes it slightly harder
☐Neutral/doesn’t affect me
☐Makes it slightly easier
☐Makes it much easier

Other comments (optional)

Please let us know if you have any suggestions on how we can improve your access to information or participation in clinical trials for ovarian cancer in Australia.

____

____

____

Is there anything else you would like to share about accessing information or participation in clinical trials for ovarian cancer?

____

____

____

Information About You:

What is your gender? (select one)

☐Female/Woman
☐Non-binary
☐Transgender
☐Other
☐Prefer not to say

What is your Australian postcode:

____

Please select your highest level of education qualification achieved: (select one)

☐Less than Year 12 or equivalent
☐Completed Year 12 or equivalent
☐Trade or technical certificate or diploma
☐University undergraduate degree
☐Postgraduate/higher university degree

Which ethnic group/s do you identify with?

Please note that ethnicity is a subjective and self-defined concept. Your ethnic identity might reflect your ancestry, cultural heritage, values, traditions, rituals, language or religion. There is no right or wrong answer.

☐Australian (including Aboriginal Australian and mixed ethnicity such as British-Australian, Asian-Australian)

☐Other (Branch to question below)

☐Prefer not to say

Branch "Other" – What ethnic group do you identify with?

____

What is your preferred language to read health information? (select one)

☐English
☐Mandarin
☐Arabic
☐Vietnamese
☐Cantonese
☐Punjabi
☐Other (Branch to question below)

Branch "Other" – In what other language do you prefer to read health information?

____

Are you of Aboriginal origin, Torres Strait Islander origin, or both? (select one)

☐Yes, Aboriginal
☐Yes, Torres Strait Islander
☐Yes, both Aboriginal and Torres Strait Islander
☐No
☐Prefer not to say

How long ago were you <u>first diagnosed</u> with ovarian, fallopian tube or peritoneal cancer? (select one)

☐Up to 12 months ago
☐Between 1 and 2 years ago
☐Between 2 and 3 years ago
☐Between 3 and 4 years ago
☐Between 4 and 5 years ago
☐5 years ago or more

Please select the sub-type/s of cancer you were diagnosed with: (click all that apply)

☐High grade serous
☐Low grade serous
☐Rare sub-type (Branch to question below)
☐Other (Branch to question below)
☐I'm unsure/I can't remember

Branch "Rare sub-type" and "other" - What type of ovarian cancer were you diagnosed with?

____

Through which type of healthcare service did you receive cancer care?

☐Public
☐Private
☐Both public and private
☐I don't know/I can't remember
☐Other (Branch to question below)

Branch "Other" – Through which other type of healthcare service did you receive cancer care?

____

Thank you so much for taking the time to complete this survey.
