# Supplementary material for: Listening to Australians with ovarian cancer: a cross-sectional survey investigating clinical trials awareness, information access and participation
Source: Support Care Cancer. 2026 Mar 22;34(4):350. doi: 10.1007/s00520-026-10586-1 (PMC13005823; doi:10.1007/s00520-026-10586-1)
Supplement: Supplementary file 3 — (PDF 428 KB) [file 520_2026_10586_MOESM3_ESM.pdf]

SUPPLEMENTARY FILE 3

RECRUITMENT MATERIALS

Title:

Listening to Australians with ovarian cancer: a cross-sectional survey investigating clinical trials awareness, information access and participation

Journal

Supportive Care in Cancer

Authors:

Natalie Williams; Yeh Chen Lee; Hayley Russell; John Andrews; Won Sun Chen; Bridget Bradhurst

Corresponding author: Natalie Williams, Ovarian Cancer Australia; Curtin University
natalie.f.williams@curtin.edu.au

RECRUITMENT EMAIL

On ,Tue Sep 24 2024 10:00:27 GMT+1000 (Australian Eastern Standard Time), Ovarian Cancer Australia <support@ovariancancer.net.au> wrote:

---------- Original Message ----------

Australian women with ovarian cancer have told us they want to know more about the availability of clinical trials. Clinical trials are a way for scientific researchers to test and measure new treatments and the way people are provided with cancer care.

OVARIAN CANCER AUSTRALIA

OVARIANCANCER.NET.AU

OVARIAN CANCER AUSTRALIA

OVARIANCANCER.NET.AU

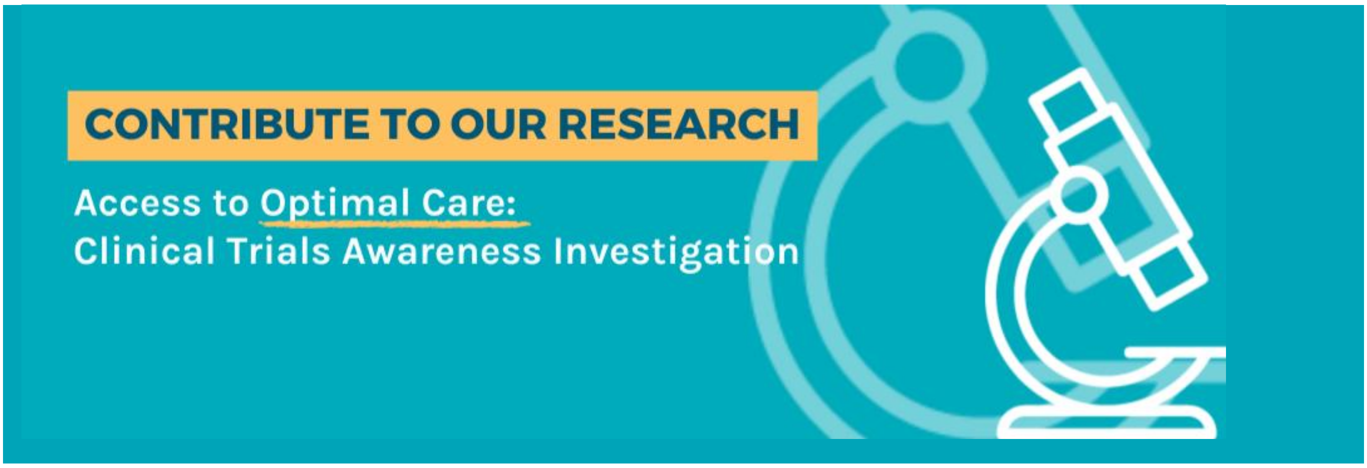

Australian women with ovarian cancer have told us they want to know more about the availability of clinical trials. Clinical trials are a way for scientific researchers to test and measure new treatments and the way people are provided with cancer care.

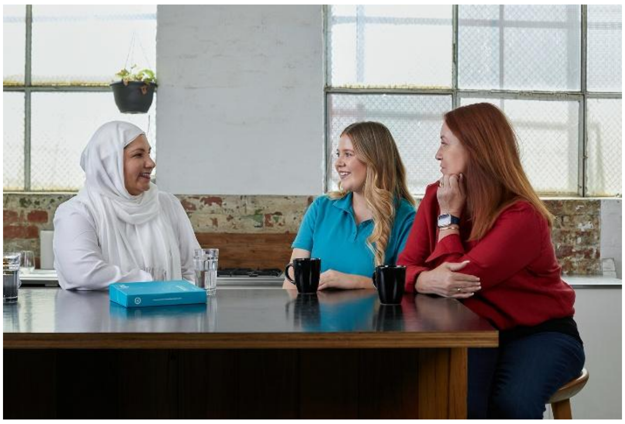

Ovarian Cancer Australia has received support from the NSW Government to develop information resources to help improve knowledge and awareness of clinical trials for ovarian cancer. To do this, we need to know more about what helps and challenges people with ovarian cancer to find out about clinical trials.

If you are a person who has had a diagnosis or recurrence of ovarian, fallopian tube or peritoneal cancer within the past five years and you live in Australia, we invite you to complete our survey.

It is expected the survey will take approximately 15-20 minutes to complete and you can do this at a time that is convenient to you. During the survey, questions will be asked about your basic demographic information, awareness of clinical trials, factors that help and challenge access to information about clinical trials and referrals, and preferred sources of information.

I want to be involved

I want to learn more

This study has been approved by the Curtin University Human Research Ethics Committee (HREC) (HRE2023-0637) and Child and Adolescent Health Service Health Human Research Ethics Committee (RGS0000006962). This study is funded by the Ministry of Health (NSW).

We are thankful to our advisory group members, including clinicians and women with a lived experience of ovarian cancer, for their support with this project. We look forward to sharing learnings and resources with our community in time.

For any further information or support, please don't hesitate to contact our ovarian cancer nurses via Helpline on 1300 660 334 (during business hours) or email support@ovariancancer.net.au.

OVARIAN CANCER AUSTRALIA

Ovarian Cancer Australia

Queen Victoria Women's Centre
Level 1, 210 Lonsdale St,
Melbourne VIC 3000

Unsubscribe

SOCIAL MEDIA POST

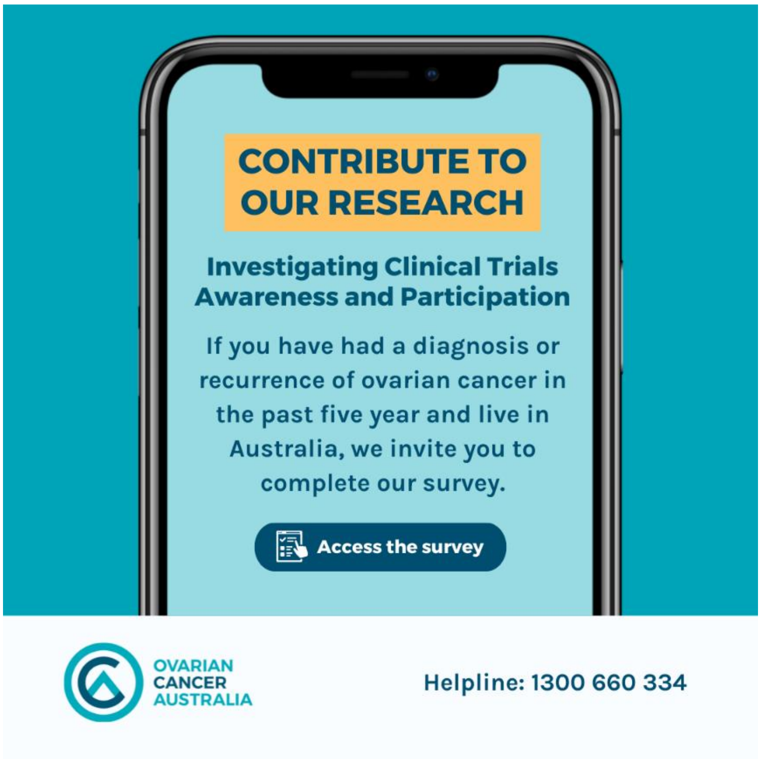

Optimal ovarian cancer care includes access to clinical trials, however there can be challenges to finding information about clinical trials for ovarian cancer. To address this, Ovarian Cancer Australia has received support from the NSW Government to develop information resources to help improve knowledge and awareness of clinical trials for ovarian cancer. The first phase of this project is to learn more about what helps, and what challenges people with ovarian cancer to find out about clinical trials.

If you are a person who has had a diagnosis of ovarian cancer within the past five years and you live in Australia, we invite you to complete our survey. It is expected the survey will take approximately 15-20 minutes to complete and you can do this at a time that is convenient to you. During the survey, questions will be asked about your basic demographic information, awareness of clinical trials, factors that help and challenge access to information about clinical trials and referrals, and preferred sources of information.

Visit our website to access the survey.

This study has been approved by the Curtin University Human Research Ethics Committee (HREC) (HRE2023-0637) and Child and Adolescent Health Service Health Human Research Ethics Committee (RGS0000006962). This study is funded by the Ministry of Health (NSW).

For any further information or support, please don’t hesitate to contact our ovarian cancer nurses via Helpline on 1300 660 334 (during business hours) or email support@ovariancancer.net.au

RECRUITMENT LETTER

Access to Optimal Ovarian Cancer Care: An Investigation of Clinical Trials Awareness and Participation
Phase 2 – Online Survey

Research Team: [Research team listed here]

Hello,

You are invited to participate in a research project that is being conducted in conjunction with Curtin University. *Curtin University Human Research Ethics Committee (record number XXX)* and [site specific ethics committee] have approved this study.

The aim of this project is to investigate factors about clinical trials awareness and access for people with ovarian cancer and find out how people with ovarian cancer prefer to receive information about clinical trials. In this phase of the study, you are invited to participate in an online survey.

It is expected the survey will take approximately 15-20 minutes to complete and you can do this at a time that is convenient to you.

Your participation in this project will remain anonymous as no names or identifying data will be collected. Only a study number will identify the information you share. All research information will be stored in password protected computer files and accessed only by the researchers named above.

Participation in this research project is voluntary, do not have to participate. If you choose to participate and change your mind, you are free to withdraw from the study at any time prior to submitting the survey responses and there will be no consequences.

If you are interested in asking questions in more detail or in participating, you can either contact the primary researcher [Name] at [email address], follow this link [link] or scan the QR code below. A copy of the Participant Information Form can be found at the start of the survey and you should read it before you agree to participate. The researcher can arrange a time to speak by phone if you prefer.

[QR code here]

Should you wish to discuss the study with someone not directly involved, in particular, any matters concerning the conduct of the study or your rights as a participant, or you wish to make a confidential complaint, you may contact the Ethics Officer on (08) 9266 9223 or the Manager, Research Integrity on (08) 9266 7093 or email hrec@curtin.edu.au.
