# Supplementary material for: Listening to Australians with ovarian cancer: a cross-sectional survey investigating clinical trials awareness, information access and participation
Source: Support Care Cancer. 2026 Mar 22;34(4):350. doi: 10.1007/s00520-026-10586-1 (PMC13005823; doi:10.1007/s00520-026-10586-1)
Supplement: Supplementary file 4 — (PDF 134 KB) [file 520_2026_10586_MOESM4_ESM.pdf]

SUPPLEMENTARY FILE 4

DATA TABLES

Title:

Listening to Australians with ovarian cancer: a cross-sectional survey investigating clinical trials awareness, information access and participation

Journal

Supportive Care in Cancer

Authors:

Natalie Williams; Yeh Chen Lee; Hayley Russell; John Andrews; Won Sun Chen; Bridget Bradhurst

Corresponding author: Natalie Williams, Ovarian Cancer Australia; Curtin University
natalie.f.williams@curtin.edu.au

Table 1. Responses to questions on receiving or finding information on clinical trials

Survey question	Yes n (%)	No n (%)	I can't remember n (%)
Have you received information on clinical trials for ovarian cancer from a health professional?	96 (37.2)	145 (56.2)	17 (6.6)
Have you looked for information on clinical trials for ovarian cancer yourself?	113 (43.8)	138 (53.5)	7 (2.7)

(n=258)

Table 2. Association with 'Have you received information on CTs from HCP?', 'Have you participated in a clinical trial for ovarian cancer?', 'Have you looked for information on CTs yourself?'

	Have you received information on CTs from HCP?			p-value
	No	Yes	Can't remember	
Length of time since diagnosis				0.009*
Up to 3 years	94 (73.4%)	50 (56.2%)	7 (46.7%)	
3 years or more	34 (26.6%)	39 (43.8%)	8 (53.3%)	
Sub-type(s) of cancer you were diagnosed with, n (%)				0.004**
High grade serous	96 (73.3%)	65 (70.7%)	5 (31.3%)	
All others	35 (26.7%)	27 (29.3%)	11 (68.7%)	
	Have you participated in a clinical trial for ovarian cancer?			p-value
	No	Yes	Can't remember	
Length of time since diagnosis				<0.001*
Up to 3 years	114 (74.0%)	31 (50.0%)	6 (37.5%)	
3 years or more	40 (26.0%)	31 (50.0%)	10 (62.5%)	
	Have you looked for information on CTs yourself?			p-value
	No	Yes	Can't remember	
Length of time since diagnosis				0.002^{+}

Up to 3 years	93 (75.0%)	55 (54.5%)	3 (42.9%)	
3 years or more	31 (25.0%)	46 (45.5%)	4 (57.1%)	
Sub-type(s) of cancer you were diagnosed with, n (%)				0.003⁺
High grade serous	79 (61.8%)	83 (80.6%)	4 (50.0%)	
All others	49 (38.2%)	20 (19.4%)	4 (50.0%)	

* Chi-Square test;

Table 3. 'If you have looked for information on clinical trials for ovarian cancer yourself, how often have you accessed the following places/people for information on clinical trials?'

		How often have you accessed the following places/people for information on clinical trials?		
		No	Yes	Can't remember
1	Australia New Zealand Gynaecological Oncology Group (ANZGOG) website / social media	34 (30.4%)	73 (65.2%)	5 (4.5%)
2	Australian and New Zealand Clinical Trials Registry (ANZCTR) website	61 (54.5%)	44 (39.3%)	7 (6.3%)
3	Cancer Council website / social media	23 (20.5%)	84 (75.0%)	5 (4.5%)
4	Ovarian Cancer Australia website / social media	9 (8.0%)	99 (88.4%)	4 (3.6%)
5	Ovarian Cancer Research Foundation website / social media	33 (29.5%)	72 (64.3%)	7 (6.3%)
6	My cancer doctor / oncologist / surgeon	16 (14.3%)	95 (84.8%)	1 (0.9%)
7	My GP (general practitioner doctor)	82 (73.2%)	29 (25.9%)	1 (0.9%)
8	A cancer nurse at my treatment centre / hospital (not Teal Support/OCA nurse)	76 (67.9%)	35 (31.3%)	1 (0.9%)
9	Ovarian cancer Australia nurse (including Teal Support nurse)	50 (45.0%)	61 (55.0%)	0 (0.0%)

10	Other people with ovarian cancer including online support groups	50 (44.6%)	61 (54.5%)	1 (0.9%)
11	Alternative health providers (i.e. Naturopath)	98 (87.5%)	13 (11.6%)	1 (0.9%)
12	Family, friends, or work colleagues	66 (58.9%)	45 (40.2%)	1 (0.9%)

Table 4. Association between Age/location/length of time since diagnosis with clinical trial participation

	3. I would have to travel further than 100km to participate in the clinical trial					p-value
	Makes it much harder	Makes it slightly harder	Neutral / doesn't affect me	Makes it slightly easier	Makes it much easier	
Age						0.021+
Under 60 years	50 (44.6%)	16 (22.9%)	22 (42.3%)	0 (0.0%)	2 (40.0%)	
60 years and above	62 (55.4%)	54 (77.1%)	30 (57.7%)	1 (100.0%)	3 (60.0%)	
Location						0.553+
Major city	97 (86.6%)	55 (78.6%)	43 (82.7%)	1 (100.0%)	5 (100.0%)	
Other	15 (13.4%)	15 (21.4%)	9 (17.3%)	0 (0.0%)	0 (0.0%)	
Length of time since diagnosis						0.060+
Up to 3 years	80 (73.4%)	39 (60.0%)	29 (55.8%)	0 (0.0%)	3 (60.0%)	
3 years or more	29 (26.6%)	26 (40.0%)	23 (44.2%)	1 (100.0%)	2 (40.0%)	

+ Fisher Exact test;
